# Successful sample preparation for serial crystallography experiments

**DOI:** 10.1107/S1600576719013517

**Published:** 2019-11-14

**Authors:** John H. Beale, Rachel Bolton, Stephen A. Marshall, Emma V. Beale, Stephen B. Carr, Ali Ebrahim, Tadeo Moreno-Chicano, Michael A. Hough, Jonathan A. R. Worrall, Ivo Tews, Robin L. Owen

**Affiliations:** a Diamond Light Source Ltd, Harwell Science and Innovation Campus, Fermi Avenue, Didcot OX11 0DE, UK; b Institute for Life Sciences, University of Southampton, Southampton SO17 1BJ, UK; c Manchester Institute of Biotechnology, The University of Manchester, Princess Street, Manchester M1 7DN, UK; d Research Complex at Harwell, Rutherford Appleton Laboratory, Harwell Oxford, Didcot OX11 0FA, UK; e School of Life Sciences, University of Essex, Wivenhoe Park, Colchester CO4 3SQ, UK; f Institute de Biologie Structurale, 71 Avenue des Martyrs, 38000 Grenoble, France

**Keywords:** serial macromolecular crystallography, XFELs, batch crystallization, vapour diffusion, micro-crystallization

## Abstract

Serial crystallography, at both synchrotron and X-ray free-electron laser light sources, is becoming increasingly popular. However, the tools in the majority of crystallization laboratories are focused on producing large single crystals by vapour diffusion that fit the cryo-cooled paradigm of modern synchrotron crystallography. This paper presents several case studies and some ideas and strategies on how to perform the conversion from a single crystal grown by vapour diffusion to the many thousands of micro-crystals required for modern serial crystallography grown by batch crystallization. These case studies aim to show (i) how vapour diffusion conditions can be converted into batch by optimizing the length of time crystals take to appear; (ii) how an understanding of the crystallization phase diagram can act as a guide when designing batch crystallization protocols; and (iii) an accessible methodology when attempting to scale batch conditions to larger volumes. These methods are needed to minimize the sample preparation gap between standard rotation crystallography and dedicated serial laboratories, ultimately making serial crystallography more accessible to all crystallographers.

## Abstract 2

Some ideas and methods on how to produce high-quality samples for successful serial crystallography experiments are presented. The methods here described are aimed at experimenters trying to convert their vapour diffusion crystallization conditions into large-scale batch micro-crystallization.

## 1. Introduction   

### 1.1. Modern serial crystallography   

**Figure 1:**
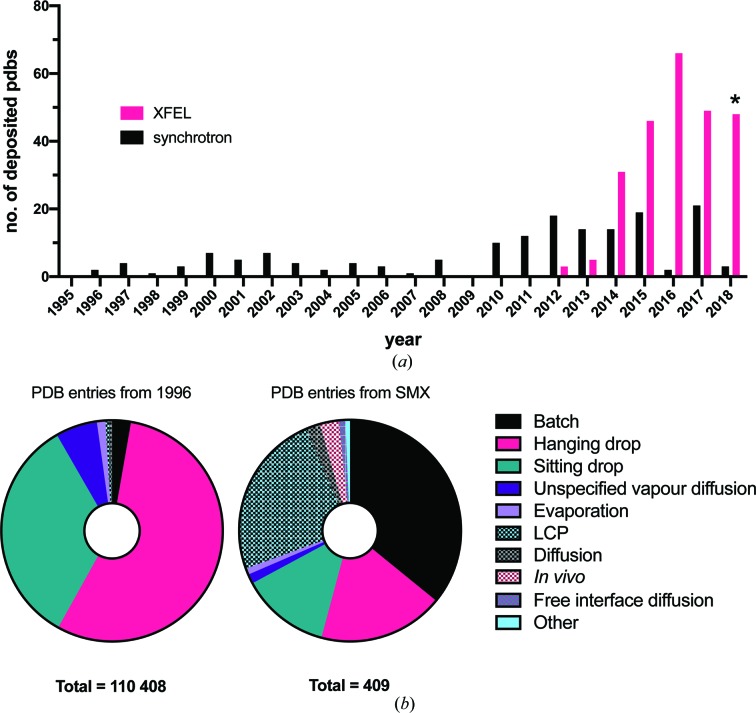
A summary of PDB depositions and crystallization methods from SMX experiments. (*a*) The frequency, plotted by year, of PDB depositions from serial experiments collected at XFEL and synchrotron light sources. PDB entries for this figure were selected on the basis of the number of reported crystals (>10), the reported radiation source and the indexing software used. The asterisk (*) indicates that the data from 2018 ▸ are not complete. (*b*) A comparison of the crystallization methods used in the PDB as a whole (left) with the serial experiments identified in panel (*a*) (right) over the same time period.

Serial macromolecular crystallography (SMX), the collection and merging of data from multiple crystals, is not new. Prior to the widespread adoption of cryo-cooling methods in the early 1990s, data sets derived from many crystals were the norm. For certain types of protein crystal, particularly those of viral capsid proteins, cryo-cooling is not possible and the merging of multiple small wedge rotations is a necessary and effective way of acquiring a complete data set (Fry *et al.*, 1999 ▸). The availability of crystals of limited size may also require the use of a microfocus beamline and a similar multi-crystal–multi-wedge approach (Evans *et al.*, 2011 ▸). However, since the development of X-ray free-electron laser (XFEL) radiation sources, the number of Protein Data Bank (PDB; https://www.rcsb.org/) depositions from SMX methods has increased [Fig. 1 ▸(*a*)]. The XFEL beam destroys the sample upon interaction (Neutze *et al.*, 2000 ▸), precluding wedged data collection, and ultimately takes serial data collection to its logical extreme, *i.e.* one image per crystal. This necessitates the need for the delivery of a steady stream of hundreds or thousands of micro-crystals into the path of the X-ray beam in order to sample reciprocal space appropriately.

The sample requirements of modern SMX delivery approaches are, therefore, radically different from those of the single-crystal or conventional multi-crystal experiments, and so are the delivery approaches that have been devised to handle them. Broadly, four sample-delivery methods exist for SMX at XFELs and synchrotrons: jets (DePonte *et al.*, 2008 ▸; Sierra *et al.*, 2016 ▸; Weierstall *et al.*, 2012 ▸; Oberthuer *et al.*, 2017 ▸), extruders (Weierstall *et al.*, 2014 ▸; Botha *et al.*, 2015 ▸; Martin-Garcia *et al.*, 2017 ▸; Weinert *et al.*, 2017 ▸), acoustic drop ejectors (ADE) (Roessler *et al.*, 2013 ▸, 2016 ▸; Fuller *et al.*, 2017 ▸) and fixed targets (Frank *et al.*, 2014 ▸; Feld *et al.*, 2015 ▸; Hunter *et al.*, 2015 ▸; Murray *et al.*, 2015 ▸; Sherrell *et al.*, 2015 ▸; Roedig *et al.*, 2017 ▸). These categories are both broad and rapidly evolving due to the relative youth of modern SMX. This means there is a lack of standardization across facilities and laboratories, presenting a confusing picture to crystallographers wanting to practise SMX. This lack of standardization also makes direct comparisons challenging [see Grünbein & Nass Kovacs (2019 ▸) ▸ for a thorough overview]. However, all have different ideal sample requirements. The aim of the experiment should dictate the type of approach used. Therefore, this will also dictate the sample requirements. The delivery method and sample should then be combined with the optimum source to ensure acceptable hit rates for the experiment to be completed within the allocated beamtime. For example, if the investigation is a time-resolved study of a light-activated enzyme–substrate complex, a fixed-target approach could be used at a low-repetition-rate source, *e.g.* SACLA, Japan (Ishikawa *et al.*, 2012 ▸) or SwissFEL, Switzerland (Milne *et al.*, 2017 ▸). The fixed targets developed at Diamond Light Source, UK, are best loaded with 10–30 µm crystals at a concentration of 5–10 × 10^5^ crystals ml^−1^ and require 100–150 µl of slurry per load (Davy *et al.*, 2019 ▸), but how can such a sample be created? What is the total sample volume that will be required during the experiment? The investigator wanting to perform this, or any, SMX experiment must grapple with these sample requirements, and it is these requirements that remain a serious impediment to the broader application of serial methods.

### 1.2. The re-emergence of batch methods   

**Figure 2:**
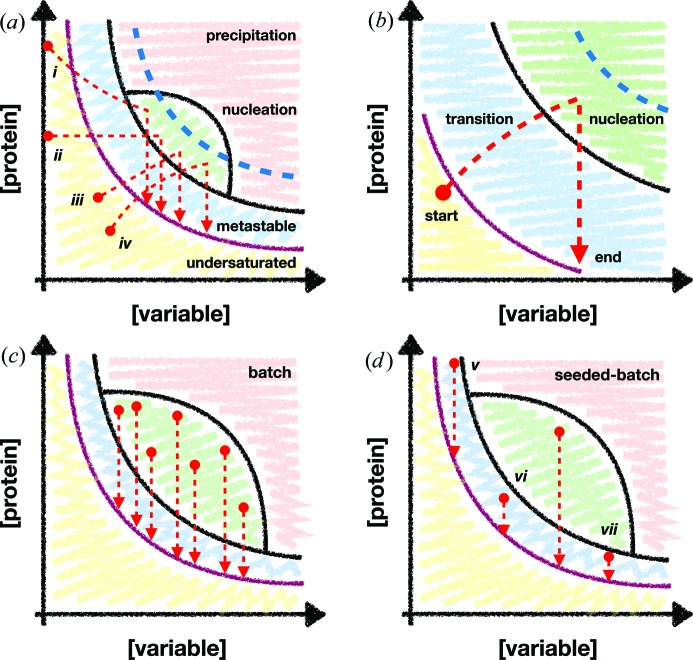
Examples of crystallization trajectories plotted onto phase diagrams. Protein concentration and a reservoir component ‘variable’ concentration are plotted on the *y* and *x* axes, respectively. The ‘variable’ could be any factor which may influence the crystallization experiment, *e.g.* PEG, salt or buffer concentration. The purple lines show the boundary of protein supersaturation [adapted from Chayen *et al.* (1992 ▸) ▸]. The red circles and arrows denote the starting and finishing points of a crystallization experiment. The regions of the diagram are labelled in panel (*a*): precipitation, nucleation, metastable and undersaturated, and these are highlighted in pink, green, blue and yellow, respectively. The blue dotted lines show the theoretical limit of nucleation-zone penetration for non-batch methods. Potential crystallization trajectories for the transitionary phase methods of free-interface diffusion (i), dialysis (ii), evaporation (iii) and vapour diffusion (iv) are highlighted. (*b*) Highlighting the trajectory of a vapour diffusion experiment. The components of the drop must transition from outside to inside the nucleation zone through some process. (*c*), (*d*) More diverse examples of batch and seeded-batch experiments, respectively. Batch experiments [panel (*c*)] are not bound by the nucleation-zone limit and can, therefore, theoretically reach every part of the region. The trajectories v, vi and vii in panel (*d*) show potential trajectories for growing large single crystals, micro-crystals and micro-crystals from a less-concentrated sample, respectively.

The large volumes of micro-crystalline samples required for SMX experiments also dictate the type of crystallization method to be used. Fig. 1 ▸(*b*) compares the relative abundance of different crystallization strategies over the same period for single-crystal crystallography and SMX. Vapour diffusion methods are significantly less popular for SMX than for single-crystal methods. SMX studies still use vapour diffusion methods but at a reduced frequency. Their place has principally been filled by batch methods, but also lipid cubic phase (LCP) and *in vivo* methods. The reason for the dominance of batch methods is perhaps not surprising, given an understanding of the crystallization process. The crystallization phase diagram [see Reis-Kautt & Ducruix (1992 ▸) ▸ and Rupp (2015 ▸) ▸ for in-depth descriptions of the kinetics and thermodynamics] highlights the problem with methods such as vapour diffusion [see Fig. 2 ▸(*a*)]. All crystallization methods apart from the batch approach rely upon a transition phase where the crystallization component concentrations must be ‘driven’ to the nucleation region by some process [Fig. 2 ▸(*b*)], *e.g.* drop equilibration (vapour diffusion).

This transition phase has several disadvantages, best exemplified by considering a vapour diffusion experiment. Firstly, the exact trajectory of the experiment is difficult to ascertain. The starting point (protein and reservoir concentrations) and finishing point (appearance of crystals) can be inferred, but not the journey between the two, *i.e.* the exact conditions that gave rise to nucleation and subsequent crystal growth are not easy to determine. Secondly, as the component concentrations within the drop mixture have to ‘move’ into the nucleation zone, it can be difficult, though not impossible,^1^ to penetrate the nucleation zone deeply [see blue dotted lines in Figs. 2 ▸(*a*) and 2 ▸(*b*)]. Transitionary phase micro-crystallization therefore requires a high rate of nucleation at the edge of the nucleation region. Finally, a successful condition in a small volume can be difficult to scale to a large volume. The exact kinetics within the drop might be essential for successful crystallization. Therefore, when scaling the experiment up to larger volumes, one must consider the additional challenge of maintaining the respective volumes of the reservoir, drop and space between.

In contrast with vapour diffusion, a batch experiment attempts to hit the nucleation zone immediately upon mixing of the protein and reservoir solutions (McPherson, 1982 ▸). The combination should create a supersaturated solution of protein which nucleates immediately. Possible batch crystallization trajectories are plotted in Fig. 2 ▸(*c*). Unlike vapour diffusion, the entire nucleation zone can be exploited in the experiment, potentially resulting in more nucleation. Scaling of the experiment is also simpler, since larger volumes of the reservoir and protein solution should produce similar results when mixed. A variant of the batch method, here called ‘seeded batch’, uses seeds (see Appendix *A*1 in the supporting information for a discussion of different types of seeds) as nucleants [Fig. 2 ▸(*d*)]. If the phase diagram is known, different regions of the metastable zone can be targeted to achieve different results. There are still questions as to the exact conditions that give rise to crystals in a batch experiment, such as how the protein and reservoir components interact in the pre-mixing time. However, these micro-scale effects will most likely be protein-condition specific and resolved naturally during the process of optimization.

The literature is not devoid of micro-crystallization examples, but a complete description of a method to make the transition from vapour diffusion to batch crystallization is currently lacking. Several papers have described techniques to identify micro-crystallization conditions using vapour diffusion. Luft *et al.* (2015 ▸) and Lee *et al.* (2018 ▸) both showed how nonlinear optics could be used to identify conditions which favour micro- (and nano-)crystalline growth in 96-well sitting-drop plates. Lee *et al.* (2018 ▸) also showed how adapting the vapour diffusion protocol using a ‘controlled evaporation’ approach increases the propensity for micro-crystallization. Both of these studies effectively focused on re-screening crystallization cocktails to find new conditions which yielded micro-crystals but did not suggest how then to scale these conditions for practical SMX. Other studies have focused on how to scale methods once a suitable condition has been identified. Ibrahim *et al.* (2015 ▸), using the case of Photosystem II, showed how different protein seed preparations and an understanding of the phase diagram could be used to find an optimum seeding protocol, whereas Kupitz *et al.* (2014 ▸) described practical large-scale methods, such as batch techniques and a novel adaptation of free-interface diffusion (FID). Darmanin *et al.* (2016 ▸) demonstrated how dynamic light scattering and powder diffraction can help test crystals prior to SMX beamtime and help ensure the sample is well optimized for the technique. However, a complete description of a method to make the transition from an initial vapour diffusion crystallization condition to a large-scale batch crystallization condition is still lacking.

This paper endeavours to shed light on how to perform this transition from nanolitre vapour diffusion crystallization to large-scale batch crystallization. This task is split into three stages: (i) optimizing crystals grown using vapour diffusion methods towards conditions appropriate for batch crystallization by finding the nucleation zone, (ii) identifying promising batch crystallization strategies by plotting a phase diagram and, finally, (iii) demonstrating a practical approach to scaling batch conditions to create the large volumes (>100 µl) of micro-crystalline slurries often needed for SMX experiments. Frequently observed problems during scaling and other crystallization tips are presented in the supporting information.

## 2. Methods   

### 2.1. PDB analysis   

#### 2.1.1. Data gathering   

The PDB analysis was conducted using data gathered on 24 July 2019 ▸. Experimental crystallization conditions were extracted from the PDB archive online. Of the 134 321 PDB entries based on crystal diffraction (X-ray, electron and neutron), 110 858 included information about how the protein was crystallized. Manual inspection of the method types led to the division of these methods into 18 broad types: vapour diffusion (sitting and hanging drop), batch, evaporation, LCP, diffusion, dialysis, counter-diffusion, *in vivo*, temperature change, FID, spontaneous growth, dilution, concentration, connected bilayer, lyophilization, centrifugal crystallization and gel acupuncture. In the few cases where the method was completely ambiguous, the crystallization method was taken from the associated publication.

#### 2.1.2. SMX analysis   

A list of PDB IDs was created by selecting SMX indicators from information contained within the PDB header. These indicators were (i) the number of reported crystals used in the experiment (>10 was used as an arbitrary indication of a serial experiment), (ii) the radiation source, *e.g.* SACLA or FREE ELECTRON LASER, and (iii) the indexing software used, *e.g. CrystFEL* (White *et al.*, 2016 ▸) or *cctbx.xfel* (Brewster *et al.*, 2018 ▸). Any PDB entry which fulfilled one or more of these conditions was considered an SMX experiment. These criteria gave a data set of 409 PDB IDs, consisting of 248 and 161 from XFEL and synchrotron light sources, respectively.

#### 2.1.3. Precipitant equilibration time analysis   

Precipitant concentration data were extracted from PDB experimental crystallization conditions for the precipitants polyethylene glycol (PEG) 8000, PEG 1000, PEG 400, 2-methyl-2,4-pen­tanediol (MPD), NaCl and (NH_4_)_2_SO_4_, comprising 5259, 1421, 10 013, 3087, 9049 and 5020 data points, respectively. Concentrations of <5% *w*/*v* or *v*/*v* and <0.5 *M* were considered likely to be only additives rather than primarily precipitants and were, therefore, excluded from the analysis. To estimate the equilibration times (90% of initial reservoir concentration at 293 K) for the different precipitant concentrations, single-phase exponential decay curves (*Prism 8*; GraphPad Software, San Diego, California, USA) were fitted to the data presented by Forsythe *et al.* (2002 ▸). Equilibration times for different precipitants were then extrapolated from the decay curves.

### 2.2. Protein preparation   

#### 2.2.1. UbiX   

UbiX protein was produced as previously described (White *et al.*, 2015 ▸). Briefly, BL21 (DE3) *Escherichia coli* cells (NEB) transformed with pNic28-Bsa4 containing *Pseudomonas aeruginosa* UbiX, codon-optimized for *E. coli*, were grown at 310 K in 22 l of Terrific Broth in a fermenter with constant aeration. The cells were induced with isopropyl β-d-1-thiogalactopyranoside (IPTG) at OD_600_ ≃ 0.8, at which point the temperature was reduced to 291 K for 18 h. Cells were harvested by centrifugation at 6000*g* for 10 min. A mass of 200 g of cells was resuspended in 50 m*M* Tris pH 8.0, 0.5 *M* NaCl, supplemented with 0.1 mg ml^−1^ DNase, 0.1 mg ml^−1^ RNase and cOmplete protease inhibitor (Sigma–Aldrich), before homogenization by French Press at 20 kpsi (1 psi ≃ 6893 Pa). The resultant lysate was clarified by ultracentrifugation at 125 000*g* for 1 h before being loaded onto 50 ml of Ni-NTA agarose (Qiagen) in a gravity flow column. The resin was washed 2 × 4 times with lysate buffer containing 10 m*M* imidazole and then 40 m*M* imidazole. Bound UbiX was then eluted from the resin using 50 m*M* Tris pH 8.0, 0.5 *M* NaCl, 0.25 *M* imidazole, before desalting into 20 m*M* Tris pH 8.0, 0.2 *M* NaCl on P-6DG resin (BioRad).

#### 2.2.2. FutA   

The FutA gene from *Prochlorococcus MED4* was inserted into a pET-24*b*(+) vector, transformed into *E. coli* BL21 (DE3) cells (NEB) and grown at 310 K in 1 l of lysogeny broth. At OD_600_ ≃ 0.4 the temperature was reduced to 291 K, and then at OD_600_ ≃ 0.6 cells were induced with IPTG and incubated for 18 h. Cells were harvested by two rounds of centrifugation at 5000*g*.

A mass of 2–4 g of cells was resuspended in IBB buffer (0.1 *M* Tris, 0.5 *M* NaCl, 1% Triton-X, 5 m*M* MgCl_2_, 10 m*M* β-mercaptoethanol). Cells were lysed by incubation with 50 mg of lysozyme and sonication, and then the inclusion bodies were washed by three cycles of 20 ml IBB buffer and centrifugation (40 min at 125 000*g* and 277 K). The inclusion bodies were dissolved in 20 ml of 0.2 *M* Tris pH 9.0, 6 *M* urea and 10 m*M* β-mercaptoethanol, incubated for 1 h at 277 K, and harvested by centrifugation for 40 min at 125 000*g* and 277 K.

FutA was refolded by rapidly diluting the supernatant into 2 l of 0.2 *M* Tris pH 9.0, 0.2 *M* NaCl, 0.4 *M* l-Arginine, 0.1 m*M* NH_4_Fe(SO_4_)_2_ and incubating at 277 K for 48 h. The refold solution was concentrated to 150 ml using an Amicon stirred cell (Merck) and dialysed overnight in 2 l of 100 m*M* Tris pH 9.0, 50 m*M* NaCl. The dialysed solution was loaded onto a 5 ml HiTrap SP XL column (GE Healthcare) equilibrated in 0.1 *M* Tris pH 9.0, 50 m*M* NaCl. The protein was eluted by the addition of 0.1 *M* Tris pH 9.0, 1 *M* NaCl and the resulting fractions containing FutA were concentrated to 80 mg ml^−1^.

### 2.3. Protein crystallization   

#### 2.3.1. UbiX   

Initial crystallization trials of UbiX used 96-well three-drop SWISSCI plates, with protein at 30, 20 and 10 mg ml^−1^ supplemented with 0.2 m*M* flavin mononucleotide (FMN). UbiX was mixed in a 1:1 ratio with precipitant, in 600 nl drops. Crystals were grown at 294 K. Multiple conditions were found to produce cubic crystals from sparse-matrix screening of UbiX; of these, 0.1 *M* MES pH 6.5, 1.6 *M* ammonium sulfate was chosen for optimization.

A phase diagram was made over two 96-well three-drop SWISSCI plates, varying the ammonium sulfate concentration on the horizontal axis from 0.1 to 3.0 *M* with constant 0.1 *M* MES pH 6.5. The UbiX concentration was varied along the vertical axis and split over the two plates, starting from 5 mg ml^−1^ and increasing to 80 mg ml^−1^ in 5 mg ml^−1^ increments. Each concentration of UbiX was supplemented with 0.2 m*M* FMN prior to crystallization. Two 300 nl drops per well were set up, one drop containing a 1:1 protein-to-precipitant ratio and the other containing a 3:2:1 ratio of protein to precipitant to seeds. The seed stock was made from the initial condition identified in the sparse-matrix screen; crystals from five drops were added to 50 µl of reservoir solution and crushed using a Hampton Seed Bead, with 90 s of vortexing.

#### 2.3.2. FutA   

To grow seed crystals of FutA, 52 mg ml^−1^ FutA solution was crystallized in 24-well XRL plates (Mol­ecular Dimensions) containing 0.2 *M* NaSCN and varying concentrations of PEG 3350 from 10 to 20%(*w*/*v*). FutA and precipitant were mixed in a 1:1 ratio in 1 µl drops and the plate incubated at 294 K. FutA seed stocks were made by pooling ten 1 µl drops, adding 40 µl of 20% PEG 3350 and vortexing the solution with a Hampton Seed Bead for 180 s. A phase diagram was created as described in Section 2.4. The FutA and precipitant concentrations were varied between 18.75 and 80.00 mg ml^−1^ in eight steps, and between 5 and 40%(*w*/*v*) in 12 steps, respectively, with a constant concentration of 0.2 *M* NaSCN applied to all reservoir solutions.

For batch crystallization, FutA (52 mg ml^−1^), FutA seed stock and crystallization buffer were mixed in a 1:1.5:1.5 ratio. Crystallization buffer [38%(*w*/*v*) PEG 3350, 0.25 *M* Tris pH 7.1] was mixed with FutA solution and vortexed for 3 s. FutA seeds, diluted 1:100 in 20%(*w*/*v*) PEG 3350, were added to the crystallization solution, which was then vortexed for 10 s. This mixture was incubated at 294 K for approximately 1–2 h and the micro-crystals were used fresh for any subsequent experiments.

### 2.4. Phase diagram crystallization experiments   

With the exception of UbiX, all phase diagrams were generated from Greiner 96-well CrystalQuick X plates by varying the protein and precipitant concentrations over the vertical and horizontal axes, respectively. Each well contained 30 µl of the reservoir solution. Two drops of 300 nl were set up within each well, one drop containing only protein and precipitant (1:1 ratio) and the other containing protein, precipitant and seeds in a 3:2:1 ratio. The plates were incubated at 293 K in a ROCK IMAGER (Formulatrix) and imaged every 3 h for 24 h.

## 3. Transitioning from vapour diffusion to batch   

Modern serial crystallography projects focus predominantly on proteins where a crystal structure of the protein of interest is already known [though there are notable exceptions, such as Sawaya *et al.* (2014 ▸) and Colletier *et al.* (2016 ▸)]. Therefore, the vast majority of SMX projects are likely to evolve from work in which crystals can already be grown and most probably in vapour diffusion plates. This paper will focus on the process of transitioning from a small-scale (<0.2–2.0 µl) vapour diffusion experiment to a large-scale (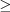
100 µl) batch protocol. Techniques such as second-order nonlinear imaging of chiral crystals (SONICC) (Luft *et al.*, 2015 ▸; Lee *et al.*, 2018 ▸) and dynamic light scattering (Abdallah *et al.*, 2015 ▸), although extremely useful in identifying conditions with micro-crystals, are not yet in the standard crystallographers’ toolbox and have, therefore, been avoided here. The tools that are described herein were chosen for either their widespread adoption or their relatively low cost, in the hope that the methods proposed are translatable to the majority of crystallization laboratories.

### 3.1. Identifying a batch-like crystallization process in a vapour diffusion crystallization condition   

The equilibration time of a sitting-drop experiment is dependent upon the composition of both the drop and reservoir volumes and on the volume of air in the well (Luft *et al.*, 1996 ▸; Forsythe *et al.*, 2002 ▸; Martins *et al.*, 2008 ▸). An understanding of the effect that drop components have on the drop equilibration time and knowledge of when crystals appear give an insight into the major crystallization ‘force’, *i.e.* the process that is driving crystallization, within the drop. Does protein crystallization require the equilibration of the drop components to find the nucleation zone (vapour diffusion), or is the nucleation zone found simply by mixing the drop components, with crystallization beginning immediately (batch)?

**Figure 3:**
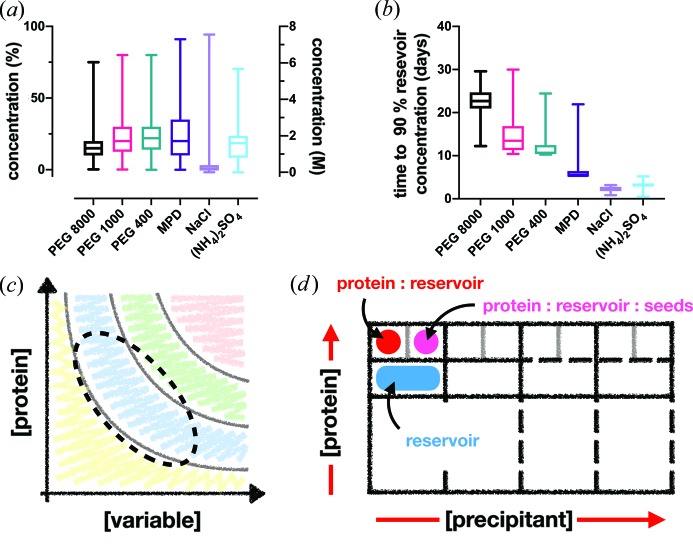
Manipulating vapour diffusion crystallization conditions into batch. (*a*), (*b*) Box-and-whisker plots of the submitted PDB precipitant concentrations from vapour diffusion crystallization experiments and their extrapolated equilibration times (time to 90% reservoir concentration), respectively. The diffusion times were calculated from data given by Forsythe *et al.* (2002 ▸) ▸. (*c*) The archetypal phase diagram, showing the likely area where the majority of vapour diffusion crystallization experiments begin (dotted line). (*d*) A design of a crystallization experiment in a two-drop 96-well sitting-drop plate to determine the phase diagram of the protein–precipitant mixture. One drop contains only protein and reservoir solution and the other contains protein, reservoir and seed solution, allowing the plotting of the nucleation and metastable zones, respectively.

Fig. 3 ▸(*a*) shows the principal precipitant concentrations for all vapour diffusion experiments that were reported and could be extracted from PDB entries (for example, https://www.rcsb.org/pdb/explore/materialsAndMethods.do?structureId=100d) using either PEG (400, 1000 or 8000) and/or salt-based [NaCl and (NH_4_)_2_SO_4_] precipitants. Calculated equilibration times [extrapolated from principal precipitant concentrations using values calculated by Forsythe *et al.* (2002 ▸)] are shown in Fig. 3 ▸(*b*). Although these equilibration times are based upon mono-component solutions where equilibration has been shown to be longer than in more complex mixtures (Luft & DeTitta, 1995 ▸), the broad trend is still applicable. The fact that longer equilibration times are observed for PEG precipitants means that, if crystals appear rapidly (within the first 12–24 h of a vapour diffusion experiment), then although the drop equilibrium will already be shifting, the crystallization ‘force’ is still more likely to be ‘batch like’ than pure vapour diffusion. A batch-like process may also be true for rapidly appearing crystals under salt-based conditions; however, if crystals appear after 4–5 days, the drop equilibration is probably complete, meaning that, again, the crystallization force is more likely to be batch like.

Knowledge of how crystallization time and drop equilibration intersect has two implications. Firstly, by limiting (in the case of PEG precipitants) or lengthening (generally, in the case of salt precipitants) the time horizon of a vapour diffusion experiment, vapour diffusion crystallization conditions can be screened for batch-like conditions. Secondly, and very practically, the hunt for batch-like conditions can be done in small-volume (200 nl) 96-well sitting-drop plates, which are already widely used and integrated into most crystallization facilities.

At this point, it is also worth mentioning microbatch methods (Chayen *et al.*, 1990 ▸, 1992 ▸), which were initially designed to make batch crystallization more compatible with robotic methods. This paper focuses on using vapour diffusion tools to make the conversion into batch as these are generally more widely used than microbatch, but the conversion could also be made using microbatch techniques instead (Chayen, 1998 ▸). However, successfully growing crystals in microbatch plates is not necessarily a marker of a batch-like condition, *i.e.* hitting the nucleation zone immediately upon mixing protein and precipitant. This is because evaporation occurs through the oil covering the microbatch drop, changing the concentration of crystallization solution components (Chayen, 1998 ▸). Indeed, this evaporation can even be exploited to aid crystallization by tailoring the mixture of mineral oils used to cover the crystallization drop to allow more evaporation (D’Arcy *et al.*, 2003 ▸). Ultimately, this evaporation process means that crystals grown in a microbatch experiment may suffer the same transitionary phase problems as described for vapour diffusion crystallization, making it difficult to pinpoint the nucleation zone and the exact concentration of components in the condition required for crystal nucleation. Nevertheless, crystallization time in microbatch, like in vapour diffusion, could very likely act as a guide to help identify the nucleation zone, but it might add a step in the process of transitioning to true batch crystallization.

### 3.2. Optimizing for batch crystallization   

Upon examination of the crystallization time, if the protein of interest already crystallizes in a batch-like process, the nucleation and metastable regions of the condition can be explored (see Section 4). If the crystallization condition is not already batch like, the crystallization time can act as a rough guide as to how far a given condition is from the nucleation region. Therefore, by varying drop component concentrations and using either a shorter (PEG-based conditions) or a longer (salt-based conditions) crystallization time as the optimization metric, a batch-like condition can be discovered.

In theory, a true vapour diffusion experiment could start anywhere in the phase diagram. However, given the PEG and protein concentrations typically used in sparse-matrix screening, the most likely starting region is as highlighted in Fig. 3 ▸(*c*). A simple test to assess whether a vapour diffusion condition begins in the metastable region is to add seeds to the crystallization experiment. The addition of seeds to a supersaturated protein solution should produce crystals rapidly and can therefore act as a further guide in optimization. Some other potential paths are listed here and an example of the steps taken to move from vapour diffusion to a batch-like process is shown in Appendix *A*2 in the supporting information.

(i) *Multivariate experimental design.* Essentially, instead of limiting crystallization optimization to a two-dimensional approach, it is better to explore a wider region of ‘crystallization space’ by varying all components of the crystallization drop simultaneously [for a full description see Shaw Stewart & Mueller-Dieckmann (2014 ▸)]. The *XSTEP* package, from Douglas Instruments, is available to do this.

(ii) *Changing the ratio of protein to reservoir volume in the drop.* Most crystallization screening starts at a 1:1 protein-to-reservoir volume ratio. However, changing this will shift the starting point on the phase diagram diagonally, exploring different areas of the diagram.

(iii) *Sparse-matrix micro-seeding.* If the current condition is not yielding anything positive, the researcher can look for new crystallization conditions using seeds as random nucleants (Ireton & Stoddard, 2004 ▸; D’Arcy *et al.*, 2007 ▸). This method can identify novel reservoir conditions which may have a more batch-like propensity.

## 4. Exploring the metastable and nucleation regions   

Once a batch condition has been discovered, a point in the nucleation zone has also been discovered. This condition can then be used as an anchoring point to discover the size and shape of the nucleation and metastable regions of the phase diagram. Knowledge of these regions is of great utility when attempting to scale to larger volumes, since parameters such as protein concentration, crystal size and nucleation rate can be factored into the scaling arithmetic, ultimately leading to better outcomes.

### 4.1. Designing a phase diagram experiment   

Once the parameters of a batch-like experiment have been identified, it becomes straightforward to generate a phase diagram. This can be done by taking the precipitant and protein and varying their concentration to form the *x* and *y* axes of the plot. A two-drop-per-well experiment can be particularly effective [Fig. 3 ▸(*d*)]. The first drop should comprise the protein and reservoir mixture, while the second should contain a mix of protein, reservoir and seeds; a 3:2:1 ratio is a good place to start (Ireton & Stoddard, 2004 ▸) (see Section 2.4). The results from the first drop will effectively plot the nucleation region, as only protein and precipitant concentrations that hit the nucleation zone will give rise to crystals and be observed. In the second drop, drops in the nucleation and metastable region should both yield crystals, as the seeds will act as nucleants and allow crystal growth. A comparison between the two drops should allow all four regions of the phase diagram to be determined.

### 4.2. Phase diagram examples   

**Figure 4:**
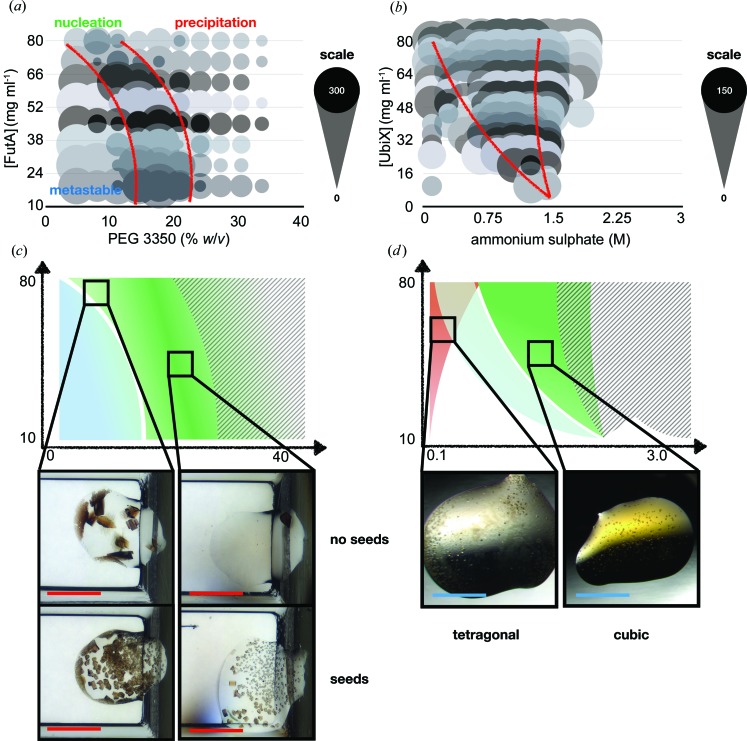
Phase diagrams for FutA and UbiX. The raw plots for *Prochlorococcus MED4* FutA and *P. aeruginosa* UbiX are shown in panels (*a*) and (*b*), respectively. The plots are based on two vapour diffusion crystallization experiments, with and without protein crystal seeds (see Section 4.1). The size of each circle corresponds to the approximate number of crystals observed in the crystallization drop. The opaque and shadowed circles show the number of crystals present from drops with no seeds and seeds, respectively. The red lines refer to the approximate boundaries between the different zones of the diagram. (*c*), (*d*) Representations of the plots shown in panels (*a*) and (*b*), respectively: darker shading indicates regions of higher nucleation, grey hatching shows drops where precipitation was visible, and the pink shading in the UbiX plot [panel (*d*)] highlights the region where a tetragonal crystal form appears. The crystallization drop images in panel (*c*) show the different levels of nucleation observed in both the seeded and un-seeded conditions. The images in panel (*d*) show the two different crystal forms of UbiX. The red and blue scale bars in the images denote 600 and 300 µm, respectively.

FutA, a periplasmic iron-binding protein associated with an Fe^3+^ uptake ABC transporter from *Prochlorococcus MED4* (Polyviou *et al.*, 2018 ▸), and UbiX, a flavin prenyltransferase from *P. aeruginosa* involved in ubiquinone biosynthesis (White *et al.*, 2015 ▸), make interesting case studies of experimentally determined phase diagrams (two further phase diagrams are presented in Appendix *A*3 in the supporting information). The FutA phase diagram [Figs. 4 ▸(*a*) and 4 ▸(*c*)], when crystallized in 0.2 *M* NaSCN and PEG 3350, is striking, because the nucleation zone does not have the expected bow shape, illustrating the importance of experimental determination of the phase boundaries. The nucleation rate was somewhat proportional to both protein and precipitant concentrations. However, protein precipitation was observed when the precipitant was further increased. The basal nucleation rate was relatively low, so a seeded-batch protocol was developed (see Section 5.1).

UbiX, when crystallized in ammonium sulfate, produced two different crystal forms as confirmed by X-ray diffraction: cubic and tetragonal (data are not shown). The tetragonal form was associated with poorer quality (lower resolution) diffraction, so the cubic form was the goal of the crystallization experiment. Fortunately, the phase diagram showed that the tetragonal and cubic crystal forms were created from relatively distinct regions of the phase diagram [Figs. 4 ▸(*b*) and 4 ▸(*d*)]. Tetragonal crystals only appeared at very low precipitant concentrations [pink shaded area in Fig. 4 ▸(*d*)], whereas the cubic form was favoured at higher precipitant concentrations. The barrier between protein precipitation and the nucleation region was relatively clearly defined: drops contained either crystals or precipitation, with both rarely occurring together. Like FutA, the nucleation rate could be influenced by precipitant concentration, but not greatly, again suggesting that perhaps a seeded-batch protocol would be more appropriate. A description of the scaling of UbiX batch crystallization to larger volumes is given in Appendix *A*4.

## 5. Scaling batch conditions to larger volumes   

Once an appropriate condition or conditions have been identified, the next task is to attempt to scale these batch or seeded-batch conditions, aiming for an eventual final volume of >50 µl but really as large as is feasible and appropriate. Scaling can be a daunting and frustrating prospect and not without reason. Protein volumes and therefore sample consumption will increase greatly. This paper cannot present any hard and fast rules, only a collection of ideas and suggestions. Like a cliff diver, at some point you have to take the plunge.

### 5.1. Optimizing crystal size and concentration   

Creating a protocol where the final size of the micro-crystals can be systematically changed is a huge advantage (Dods *et al.*, 2017 ▸). Crystal size can be optimized to the sample-delivery approach and other experimental factors, such as the required diffusion time for a ligand or the light penetration depth. Crystal concentration (crystals per millilitre) will ultimately be determined by the nucleation rate and is inversely proportional to crystal size. That is to say, the greater the level of nucleation, the greater the number of crystals that must grow from the finite amount of protein in the batch condition, so the smaller the crystals will be. However, whereas crystal concentration can be manipulated by the removal or addition of buffer after completion of the crystallization experiment, size homogeneity has to be tailored at the crystallization step. Therefore, although crystal concentration is an important consideration due to its relationship to crystal size, ultimately crystal size and size homogeneity should be the key heuristics in the scaling process as these cannot be changed (that said, see Table 2 in the supporting information for some limited advice concerning crystal crushing).

**Figure 5:**
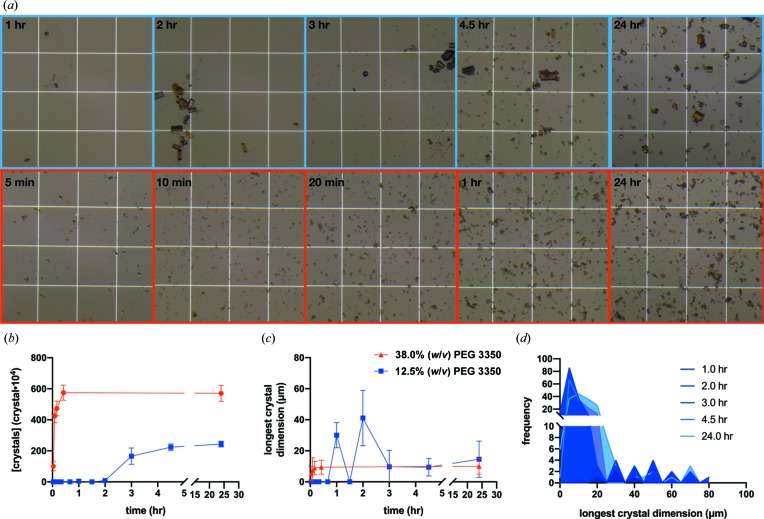
Observing a 100 µl FutA batch crystallization over 24 h. (*a*) The growth of two FutA batch crystallization experiments, the top (blue) in 0.2 *M* NaSCN, 12.5%(*w*/*v*) PEG 3350 and the bottom (red) in 0.1 *M* Tris pH 7.1, 38.0%(*w*/*v*) PEG 3350. The pictures show aliquots viewed in a hemocytometer. The white boxes in the images have dimensions of 250 × 250 µm. (*b*), (*c*) Demonstrations of how the mean number of crystals and longest dimension change over time. (*d*) A histogram of crystal size over 24 h for the 12.5%(*w*/*v*) PEG 3350 condition.

A hemocytometer [a small particle counter – Fig. 10(*e*) in the supporting information] allows the experimenter to assess a representative sample of the micro-crystals from a given crystallization experiment, allowing both their size range and the concentration to be estimated. Fig. 5 ▸ shows how this can be performed using FutA as an exemplar.

The process is as follows. During a large-scale (>20 µl) batch experiment, take regular aliquots (2.5–5.0 µl) of the crystallization experiment and view in a hemocytometer [Fig. 5 ▸(*a*)]. Ensure the batch crystallization experiment is homogeneous before taking an aliquot, and make a note of the number of crystals and their size distribution [Figs. 5 ▸(*b*) and 5 ▸(*c*)]. These data can then be used to compare different batch conditions and iterate towards an ideal protocol for a given sample-delivery approach, *e.g.* probing alterations in precipitant and/or protein concentrations or optimizing the ratios of components in the crystallization solution. It should also be noted that it is theoretically possible that the taking of these aliquots could hinder protein crystallization. However, if such effects from collecting these aliquots do occur, they have yet to be observed.

The power of this technique is shown in the case of FutA. From the initial phase diagram, 52 mg ml^−1^ of FutA solution, mixed in a 1:1 ratio with 0.2 *M* NaSCN, 12.5%(*w*/*v*) PEG 3350, was selected as a starting point for a seeded-batch experiment. However, as can be seen from Fig. 5 ▸ this was not ideal as the crystals were not sufficiently homogeneous in size. Although the eventual crystal concentration and size were acceptable [Figs. 5 ▸(*a*), top panel, 5 ▸(*b*) and 5 ▸(*c*)] for an SMX experiment (data are not shown), many large crystals (>40 µm) were formed early (1–2 h) in the experiment. It was only after 3 h that showers of micro-crystals were observed. This delayed start created an asymmetric size distribution [Fig. 5 ▸(*d*)], with two crystal-size populations being observed. Altering the PEG concentration did not appear to improve the homogeneity in the crystal size, but the addition of a neutral buffer did. This change was prompted by the wish to improve the durability of FutA crystals during ligand-soaking experiments. The NaSCN was exchanged for 0.1 *M* Tris pH 7.1 in the crystallization buffer because the FutA crystals dissolved in the presence of ligand and NaSCN. The exchange improved the crystal stability and also reduced the tendency for the crystals to clump together. In the presence of Tris, the propensity of the FutA to precipitate at higher PEG concentrations was also reduced. The PEG concentration could then be increased from 12.5 to 38.0%(*w*/*v*) PEG 3350. These changes reduced the size and increased the concentration of the FutA crystals obtained from the seeded-batch crystallization [Figs. 5 ▸(*a*), bottom panel, 5 ▸(*b*) and 5 ▸(*c*)].

### 5.2. Scaling up in volume   

The proposed sample-delivery mode in the SMX experiment can also dictate the final volume of the batch crystallization experiment. Some ADE and extruder delivery systems require only 20 µl of sample per load. Therefore, a final experimental volume of 100 µl, assuming that a ‘reasonable’ crystal concentration can be achieved, should be perfectly adequate for these delivery approaches. If larger volumes are required, pooling of multiple 100 µl experiments is also possible. This being the case, a step-wise volume increase from 200 nl to an approximate final volume of 100 µl could prove safest. If larger volumes of sample are required, multiple batches of 100 µl can be set up concurrently and pooled together. However, if a step-wise scale in crystallization volume has proved successful, larger volumes of 1 ml or more could also be attempted if applicable, feasible and necessary. An example of such a scaling protocol is described below. At each step, the user should assess the number of crystals and range of sizes. If these change, slight alterations should be attempted in component concentrations and/or ratios.

(i) *Increase the volume in robot-compatible plates.* Liquid-handling robots for 96-well experiments, such as the Mosquito (TTP Labtech), can aspirate volumes of up to 1.2 µl, giving an effective limit of 2.4 µl on the drop size, assuming a 1:1 protein-to-reservoir ratio. This drop size can be accommodated in some 96-well sitting-drop plates, such as the Greiner CrystalQuick [Fig. 10(*a*) in the supporting information] or the SWISSCI MRC 48-well plates. An under-oil experiment at these volumes could also be attempted, perhaps using SWISSCI under-oil or Terizaki plates [Figs. 10(*b*) and 10(*c*), respectively], the former having a maximum volume of 4 µl. The advantage of using such plates is that most are still compatible with commercially available crystallization robots and storage hotels, thus simplifying standardization and monitoring.

(ii) *Increase the drop volume to 10–20 µl.* This entails moving from robot-compatible plates into either 24-well hanging- or sitting-drop plates, PCR tubes or 0.5 ml centrifuge tubes. The crystallization experiment should be monitored in the drop or tube over 1–7 days, taking note of the crystal number and size.

(iii) *Increase the drop volume to 20–100 µl.* This is achieved by moving into 0.5 ml centrifuge tubes or 96-well chimney-well plates [Fig. 10(*d*)]. Aliquots are taken every 3–4 h to measure the crystal number and size using a hemo­cytometer [Fig. 10(*e*), and described in Section 5.1]. Gentle or even vigorous agitation may now be required, depending on the current vessel; potential mixers are shown in Figs. 10(*f*), 10(*g*) and 10(*h*).

(iv) *Increase the drop volume to 0.5–1.0 ml (if required).* If all the preceding steps are consistent, the user could try to move to 1.5 ml centrifuge tubes.

(v) *Increase the volume to 5–10 ml (if required).* The user should only attempt this if the protein can be easily produced and the delivery approach requires large (>1 ml) volumes.

### 5.3. Other tips and ideas   

Table 1 in the supporting information shows some recurrent problems that have been encountered when scaling several different proteins to large-volume batch crystallization. Some potential solutions to these problems are suggested in the table; these are by no means perfect or exhaustive but might be helpful. Other crystallization tips are listed in Table 2 in the supporting information.

## 6. Conclusions   

The aim of this paper was to suggest methods and ideas to aid in converting a vapour diffusion crystallization experiment into a larger-scale batch experiment. Given what can seem like the somewhat arbitrary whims of protein crystallization, the creation and subsequent understanding of a crystallization phase diagram is perhaps the surest way to approach these tasks. Vapour diffusion crystallization experiments can be converted into batch crystallization by understanding the role the precipitant is playing in the crystallization process and looking at the timescale of crystal nucleation and growth. Optimizing a vapour diffusion experiment in this manner allows the nucleation zone to be found, and hence the conditions for batch crystallization. Once a batch condition has been found, a phase diagram can be created. From the information in the phase diagram, batch or seeded-batch protocols can be gradually scaled to test the condition in larger volumes. This approach may ease the burden on the required protein volume and make the process of transitioning to batch crystallization more efficient. Ultimately, protein crystallization is fickle and should be assumed to fail randomly. Given this capricious tendency, the more time spent understanding the crystallization process, the greater the chance that good quality crystals will be obtained when they are required on a beamline.

## 7. Related literature   

The following additional literature is cited in the supporting information: Bergfors (2003 ▸), Berrow *et al.* (2007 ▸), Bunker *et al.* (2012 ▸), Chayen *et al.* (2006 ▸), de la Cruz *et al.* (2017 ▸), Ino *et al.* (2011 ▸), Luft & DeTitta (1999 ▸), McPherson & Shlichta (1988 ▸), Nanev *et al.* (2017 ▸), Stura & Wilson (1992 ▸), Zhang *et al.* (1996 ▸).

## Supplementary Material

Supporting information file. DOI: 10.1107/S1600576719013517/jo5052sup1.pdf

## Footnotes

1 It is potentially possible to cross the nucleation limit line using a ‘controlled evaporation’ approach (Lee *et al.*, 2018 ▸) that rapidly shifts the crystallization drop deeper into the nucleation zone, and can thereby increase the nucleation rate.
