# Supplementary material for: Successful sample preparation for serial crystallography experiments
Source: J Appl Crystallogr. 2019 Nov 14;52(Pt 6):1385–96. doi: 10.1107/S1600576719013517 (PMC6878878; doi:10.1107/S1600576719013517)
Supplement: Supplementary file 1 [file j-52-01385-sup1.pdf]

Appendix A
Supplementary Information

A.1. A brief word on seeds and seeding

[illegible]

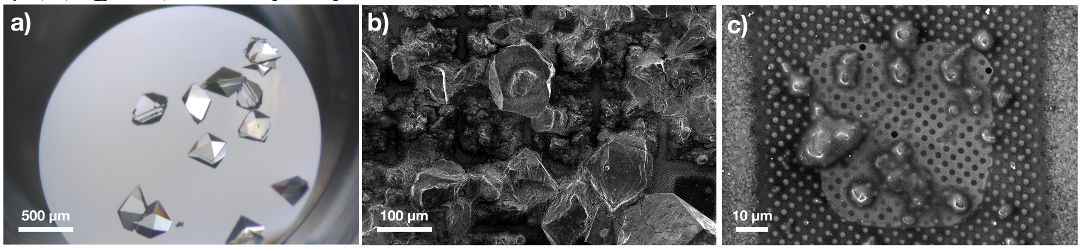

Fig. 6. **Visualisation of the steps used to generate *T. album* Proteinase K seeds. a)** shows the initial large crystals in a 24-well sitting drop plate prior to crushing with a pipette tip. **b)** and **c)** are scanning electron microscopy images of the seeds plunge frozen on electron microscopy grids after pipette crushing and three rounds of vortexing, respectively.

Seeds can be thought of as any form of protein crystals, or in fact, any scaffold molecule (see Appendix A.1.2), that when added to the protein reservoir solution mixture, act as nucleants for crystal growth (Stura & Wilson, 1992). When thinking about seeding in terms of controlling crystal size, it can be broadly divided into macro and micro-seeding protocols. Macro-seeding is where large crystals (typically > 100 µm) are used as single nucleants to grow very large crystals (not discussed further, see Lee *et al.* (2018) for a nice example). Micro-seeding is where previously grown crystals are broken up and ground down to make a cloud of nano-crystalline fragments (Luft & DeTitta, 1999; Ireton & Stoddard, 2004). These fragments retain some crystalline structure and act as a scaffold for other protein molecules to aggregate upon in an ordered manner.

There are several different ways of making a successful micro-crystal or more probably, nano-crystal seed-stock (Table 2). All involve taking larger crystals, probably grown in vapour diffusion plates, and fragmenting them with small hard spheres. Figure 6 gives an overview of the process using Proteinase K crystals from *Tritirachium album* with the aid of a scanning electron microscope (SEM). The result of the first step, crushing the seed crystals (Figure 6a) is shown in Figure 6b and the final seed stock after the three rounds of vortexing is shown in Figure 6c. A more homogeneous seed stock was shown to increase the resolution limit of crystals grown from the seeds (Dods *et al.*, 2017).

A.1.2. Heterogeneous nucleation Another method, that should be mentioned in conjunction with seeding, is heterogeneous nucleation. This method was first demonstrated by McPherson & Shlichta (1988) and has since evolved such that there are now a range of small molecule molecular scaffolds (Ino *et al.*, 2011) and porous materials (Chayen *et al.*, 2006; Nanev *et al.*, 2017) that have been shown to be effective protein crystal nucleants. In the context of micro-crystal growth, if a given protein's propensity to nucleate was increased by the presence of one of these materials, the material could theoretically be used in the same manner as a protein crystal seed stock, enabling the rate of nucleation to be directly controlled. This has not been attempted in this work but could, in theory, be successfully employed as an alternative to protein crystal seeded-batch.

A.2. Example optimisation of enhanced Green Fluorescent Protein from vapour diffusion to batch

Enhanced Green Fluorescent Protein (eGFP), adapted to increase its fluorescent emission and codon optimised for mammalian expression from *Aequorea victoria* (Zhang *et al.*, 1996), was gradually optimised from vapour diffusion to batch using the crys-

tallisation time as the key heuristic (Figure 7). This optimisation was performed in 96-well Greiner CrystalQuick™ 3-drop, 96-well plate (Figure 10a) using a reservoir volume of 90 µL and a different drop ratio in each of the wells: 1:1, 1:2 and 2:1, protein to reservoir solution. The protein was concentrated to 55 mg/mL post purification in 50 mM HEPES pH 8.0, 0.1 M $MgCl_2$ and crystals were observed after 20 days from a condition in a MORPHEUS (Molecular Dimensions) sparse matrix screen (Figure 7a).

However, the complexity of condition (90 mM Nitrate Phosphate Sulfate (NPS) additives, 0.1 M Tris/BICINE pH 8.5, 37.5 % (v/v) Precipitant Mix 4) and the appearance of salt crystals within the drop, made optimisation challenging. The protein was exchanged into 50 mM HEPES pH 8.5, 0.15 M NaCl and re-screened. A hit was now observed after 14 days in JSCG+ (Molecular Dimensions): 0.1 M BisTris Propane pH 6.5, 0.2 M NaBr, 20 % (w/v) PEG 3350 (Figure 7b). These crystals could be recreated (Figure 7c) and the crystallisation time reduced to 8 days by increasing the PEG concentration and pH to 21.6 % and 7.15, respectively.

No precipitation was visible when using an eGFP concentration of 55 mg/mL, so the concentration was increased to 120 mg/mL. Interestingly, crystals did grow at this protein concentration, but only in the 1:2 protein to reservoir drop and with the pH and PEG concentration reduced to 6.5 and 18.2 %, respectively. Figure 7d and e shows eGFP micro-crystal growth after 24 and 48 hr, respectively. To better view these crystals, Figure 7f shows an electron micrograph of the crystals from Figure 7e. The crystals are approximately 0.5 nm wide with the longest dimension of between 5 and 30 µm. Although these crystals still require further optimisation for SMX, this is an example of how to find the nucleation zone and optimise towards batch.

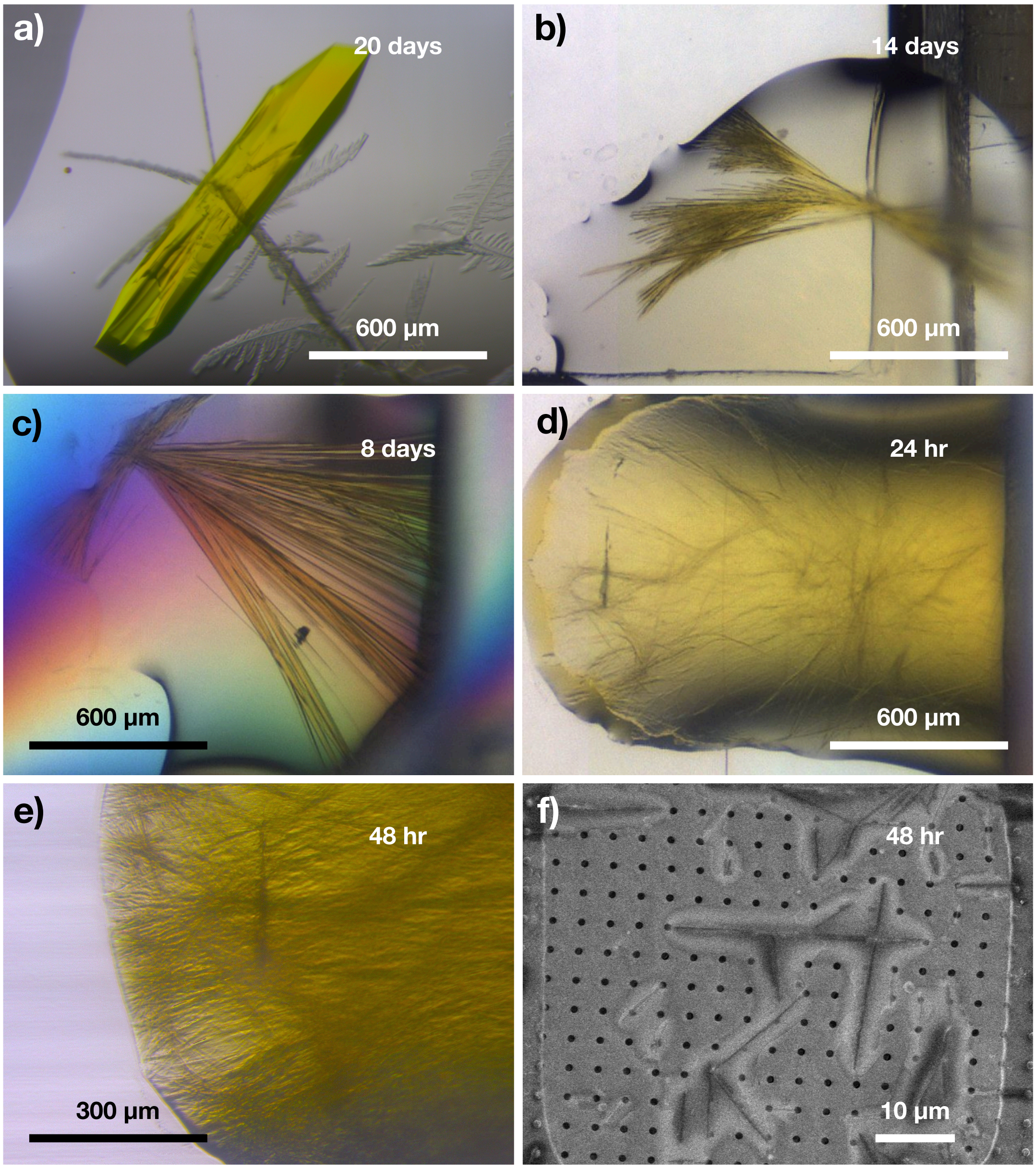

Fig. 7. **The steps taken to move from an initial vapour diffusion hit to a batch-like crystallisation process for eGFP. a)** to **e)** show images of eGFP crystals in Greiner CrystalQuick™ 96-well plates. The crystals in **f)** were removed from the well in **e)** and vitrified on an electron microscopy grid.

A.3. Insulin and thermolysin phase diagrams

The phase diagrams of bovine insulin crystallised in 0.1 M BisTris Propane pH 7.5, 0.2 M KI, PEG 3,350 and thermolysin from *Geobacillus stearothermophilus* crystallised in 0.2 M NH_4Cl, PEG 3350, nicely illustrate the importance of crystallisation rates when designing large scale batch protocols (Figure 8).

The nucleation rate of insulin appeared to be proportional to both the concentration of insulin and the concentration of PEG 3,350 (Figure 8a and c) and did not appear to be affected by the presence of seeds except at 25 mg/mL. These data suggested that in the context of a micro-crystallisation starting point, both seeded-batch and batch crystallisation techniques were viable strategies and that high protein and PEG concentrations would be necessary.

Thermolysin, by comparison, had a larger nucleation zone but a relatively low nucleation rate (Figure 8b and d). Adding seeds to the crystallisation condition gave near-perfect nucleation parity across all drops in the nucleation and metastable zones. This suggested that thermolysin nucleation in this condition was slow and that the addition of seeds could overcome any nascent nucleating power that would otherwise exist. It was also apparent that increasing the protein concentration in solution had little to no effect on the number of nucleation events. Therefore, when considering micro-crystallisation conditions for thermolysin from this condition, a seeded-batch method would be advisable and a low protein concentration should yield equally good results as a high concentration. In this case, understanding of the phase diagram would allow for greater economy of the protein sample.

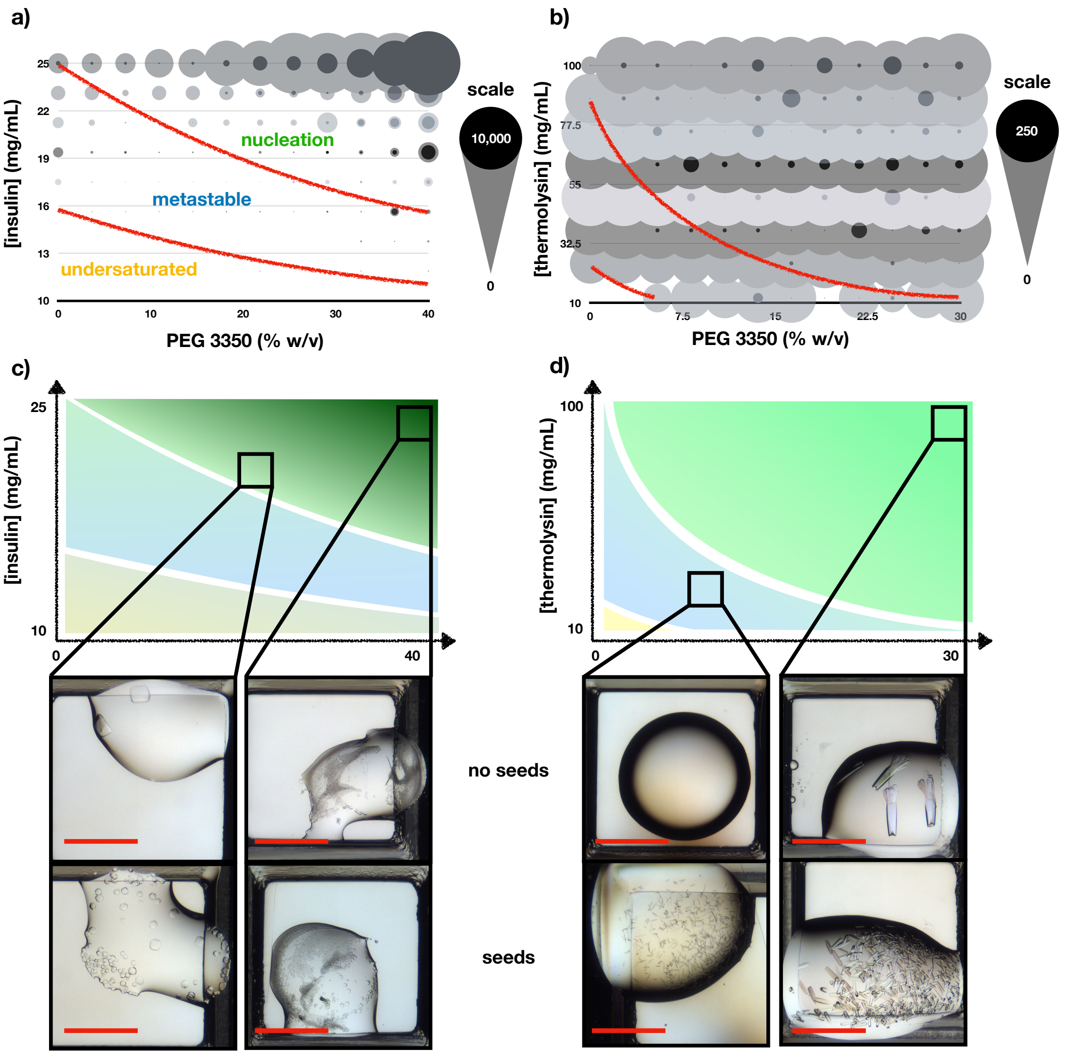

Fig. 8. **Phase diagrams for insulin and thermolysin.** The raw plots for bovine insulin and *G. stearothermophilus* thermolysin are shown in **a)** and **b)**, respectively. The plots are based on two 96 condition crystallisation experiments, with and without seeds (see § 3.1). The size of each circle corresponds to the approximate number of crystals observed in the crystallisation drop. The opaque and shadowed circles show the number of crystals present from drops with no seeds and seeds, respectively. The red lines refer to the approximate boundaries between the different zones of the diagram. **c)** and **d)** are representations of the plots shown in **a)** and **b)**, respectively where darker shading indicates regions of higher nucleation or blurring between the region division. The crystallisation drop images that are shown highlight the differing scales of nucleation between different regions of the plot for insulin and thermolysin. The red scale bars in the images denote 600 μm.

A.4. UbiX scaling

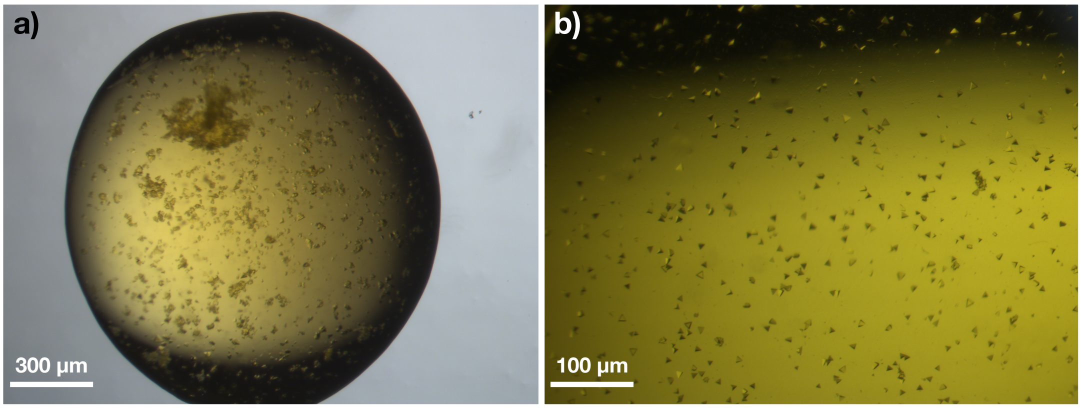

Fig. 9. **Scaling UbiX crystallisation to 100 µL. a)** and **b)** illustrate the final scaling challenge encountered for UbiX when scaled to 100 µL. **a)** show the crystal clumping observed when using seeds and **b)**, no seeds.

Attempts to scale UbiX using a seeded-batch protocol for larger volumes proved challenging. The seeded crystals tended to clump together (Figure 9a) and could not be separated without damaging the integrity of clumped crystals. Therefore, a straight batch protocol in 96-well, chimney-well plates (see Figure 10d) was created. The final crystal concentration was lower than the seeded method, approximately 6×10^4 crystals/mL. However, the final crystals remained small (10 – 15 µm) and the clumping was significantly reduced (Figure 9b), so ultimately, the straight batch protocol proved more successful.

A.5. Useful tools

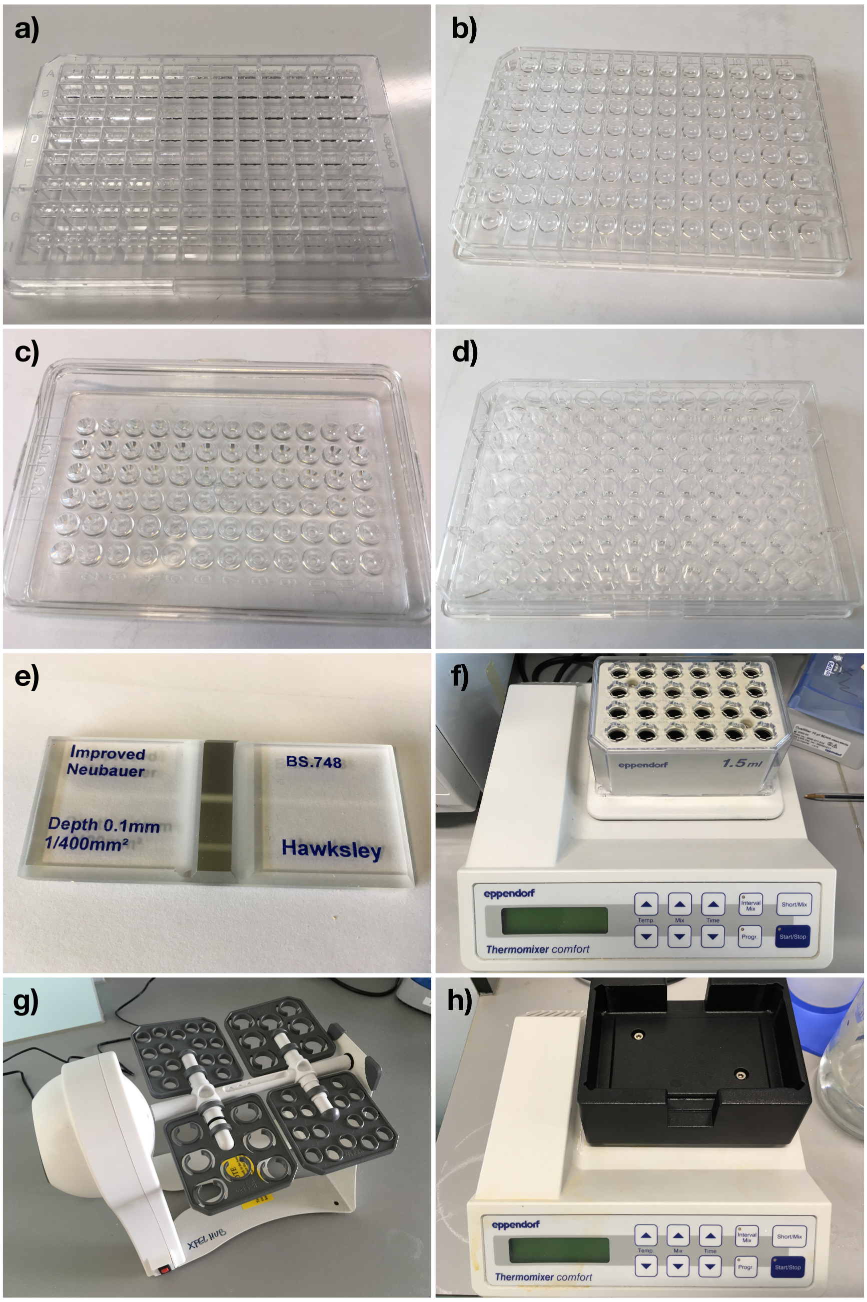

Fig. 10. **Useful tools to scale small volume batch crystallisation conditions to larger volumes. a)** 96-well Greiner CrystalQuick™ 3-drop, **b)** SWISSCI MRC under-oil plate, **c)** Greiner 72-well Terizaki microplate, **d)** 96-well F-Bottom (chimney well) microplate, **e)** Hawksley hemocytometer, **f)** Eppendorf thermomixer with centrifuge tube adaptor, **g)** Thermo Scientific™ tube revolver/rotator, **h)** Eppendorf thermomixer with plate adaptor.

IUCr macros version 2.1.1: 2009/05/11

A.6. Scaling tips

**Scaling problem**	**Possible solution**
Heterogenous crystal size	In larger batch volume crystallisation, where nucleation can happen more slowly, crystals can often sink and stick to the side or bottom of the crystallisation vessel. If nucleants become buried, they are unable to grow, whilst non-buried crystals are exposed to more free protein and grow too large. Vortexing, either gently ($10 - 20$ rpm) or rapidly (> 1000 rpm) can help to alleviate this problem. The precise level of agitation will be protein and condition-specific. Anecdotally, smaller (< 200 µL) and larger (> 500 µL) volumes might require more and less agitation, respectively.
Crystal clumping	Crystals in scaled batch conditions can clump together and/or stick to the bottom of the crystallisation vessel post-growth. Often, gentle pipetting of the crystallisation solution around the vessel is enough to remove these sticky crystals. However, if they refuse to move, gently sonicating the container in a sonication bath for $10 - 30$ s can help the crystals to lift off the sides of the vessel or break apart without apparent adverse effects to the crystals.
Degradation of seed stocks	If a seeded batch protocol is being used, a plan for the creation and storage of seed-stocks is required. A seed-stock, once created can be stored at -20 °C however, repeated freeze-thaw has been found to erode the potency of the stock and therefore reduce the level of nucleation observed. To overcome this, it can be useful to store seed-stocks as aliquots, such that seeds only undergo a single cycle of freeze-thaw.

Variations in temperature and humidity	It has been noted that the success of a batch protocol can often depend on its location. A protocol that has been successful in one laboratory can often fail when it is applied in a different laboratory. The reason for this perhaps, is that batch protocols are often conducted on the lab bench as opposed to the more controlled environment of an incubator. Subtle differences in temperature and humidity between laboratories exist that lead to different results. If a well-defined protocol is failing in different locations, the crystallisation could be sensitive to these changes. An answer here is to use heat blocks (Figure 10f) or humidity tents to control temperature and humidity as much as possible.
Micro-crystals grow too large	Quenching, *i.e.* stopping the progress of the crystallisation experiment, can be useful when the crystal size goes beyond the limit of the sample delivery approach being used (Kupitz *et al.*, 2014). Although perhaps not ideal, as quenching by definition means that there is wasted protein in solution, the technique is definitely useful if a crystallisation protocol is in all other respects satisfactory and a pragmatic appraisal suggests that 'this is good enough'.

Table 1: **Frequently observed problems when optimising and scaling batch crystallisation conditions for SMX.**

Other tips and tricks	Notes
Varying protein & reservoir volume ratios	This idea is referred to in general terms in § 2.1 and the eGFP example in Appendix A.2 is a nice example. The standard drop ratio of 1:1 protein to reservoir solution is a variable that, when altered, can yield powerful results. In § 2.1 a suggestion is made to vary this at the stage of finding batch versions of a sitting-drop experiment. This is ideal, as it limits the sample burden to a stage in the process where 200 nL experiments are being performed. However, when scaling to larger volumes, tweaks in the ratio (anecdotally less protein, more reservoir) can help push the crystallisation experiment to greater levels of nucleation.
Just add seeds	This, in many ways, is perhaps the easiest path to micro-crystallisation. For many vapour diffusion crystallisation conditions, the condition starts in the metastable region, *i.e.* the protein is already supersaturated but unable to nucleate. The vapour diffusion equilibration time would allow the drop to slowly migrate to the nucleation zone and spontaneous nucleation, however, the addition of seeds negates this and dictates the level of nucleation. The danger of solely relying on this approach is that any knowledge about the manner of protein crystallisation is lost, and therefore any understanding of the limits of the phase diagram regions. However, just adding seeds can be very quick and effective.
Standardising seed stocks	The key here is to standardise the protocol for creating the crystals which are going to be made into seeds, such that there is little variation between the size and number of crystals in the wells. In the authors' opinion, the best way to do this is to grow the seed crystals in a vapour diffusion plate and then collect the crystals from a standardised number of drops each time the seed stock is made. Variations in crystals size and number between drops will average out across the pooled drops and if a more concentrated seed-stock is required, the number of pooled drops can be easily increased. The creation of a more concentrated stock which can then be diluted will also lead to greater reproducibility.

Serial seeding	Serial seeding involves multiple rounds of seeding where crystals are produced using seeds that originate from a preceding crystal growth, *i.e.* crystals produced using an initial set of seeds are subsequently used to make a second set of seeds *etc.* By undertaking multiple rounds of serial seeding both crystal homogeneity and diffraction quality can be improved (Bergfors, 2003; Bunker *et al.*, 2012; Dods *et al.*, 2017; Ibrahim *et al.*, 2015).
Seed homogenisation	The standard method of producing crystal seeds is by vortexing larger crystals in a centrifuge tube containing a single Seed Bead™ (Hampton Research - Hampton now sell four different seed bead kits: PTFE, steel, ceramic and glass) or multiple small beads. This breaks up the crystals into smaller fragments, yet these fragments are unlikely to be completely homogeneous in size and some large fragments are likely to remain. To minimise the presence of larger fragments, the seed stock can be filtered or centrifuged at 2,000 rpm for $20 - 30$ s in a benchtop centrifuge.
Testing micro-crystal diffraction	It is essential that the diffraction quality of any micro-crystals is tested before the serial experiment, particularly if the experiment is happening at an XFEL. If a crystal does not diffract at a synchrotron, it is unlikely to diffract at an XFEL.
Standard protein stock	Given the large volumes of protein required throughout the development of a large-scale, batch crystallisation protocol, time spent maximising the efficiency of the protein preparation protocol is even more necessary. Variations in the protein preparation can affect the crystallisation significantly, therefore, the more available protein which comes from identical preparations, the greater the chance of minimising differences in protein solution.

Crystal crushing	If the crystallisation experiment does not respond well to quenching or other methods to reduce the crystal size, it is possible to crush larger crystals (> 50 µm) in order to make a suitably sized crystal slurry (Dods *et al.*, 2017; de la Cruz *et al.*, 2017)[1]. However, this is not ideal and crushing should perhaps be used as a last resort. The authors have not extensively tried this approach, on a single occasion when it worked successfully, a very short ($< 1 - 2$ s) vortex of the crystalline slurry with a Seed Bead™ (Hampton) was all that was required. Too long or too short a vortex and the crystals did not mount successfully. When the crushing was successful, there was a corresponding drop of $0.2 - 0.5$ Å in diffracted resolution compared to an uncrushed crystal which diffracted to 2.5 Å (data are not shown).

Table 2: **Some other tips and ideas on how to scale batch crystallisation conditions to larger volumes.**

Appendix B
Supplementary Methods

B.1. Protein preparation

Bovine insulin (I5500, Sigma-Aldrich) was solubilised in 50 mM sodium hydrogen phosphate pH 10.5, 10 mM ethylenediaminetetraacetic acid (EDTA), to a final concentration of 25 mg/mL. Thermolysin from *G. stearothermophilus* (T7902, Sigma-Aldrich) was solubilised in 20 mM MES pH 6.0, 45 % (v/v) DMSO to a final concentration of 100 mg/mL. Proteinase K from *T. album* (P2308, Sigma-Aldrich) was solubilised in 25 mM Tris pH 7.5 to a final concentration of 50 mg/mL. These solutions

[1] In the case of Dods *et al.* (2017), they specifically showed how crystal-crushing, followed by rounds of seeding could produce a viable sample - essentially a seeded-batch protocol - and de la Cruz *et al.*(2017) were making protein samples suitable for electron diffraction experiments.

were then filtered through a 0.2 µm centrifugal filter (EMD Millipore), flashed-cooled in liquid nitrogen and stored at −80 °C until used.

eGFP (UniProtKB: C5MKY7) was cloned into pOPINF[2] (Berrow *et al.*, 2007), and expressed in *E. coli* strain BL21-DE3. Transfected cells were grown at 37 °C with constant aeration until an $OD_{600} \approx 0.5$. At which point the temperature was reduced to 25 °C and cells were induced by the addition of IPTG. Cells were harvested after approximately 16 hr by centrifugation and lysed by a combination of freeze/thaw and cell disruption (Constant Systems). The recombinant protein was purified using Ni-immobilized metal affinity chromatography, firstly to separate fusion protein from cell lysate and then, post-His-tag cleavage, to remove the tag, protease and any remaining lysate material. Protein was then passed through a 1660 Superdex S75 (GE Healthcare) gel filtration column equilibrated in 50 mM HEPES pH 8.5, 0.15 M NaCl, concentrated, flash cooled in liquid nitrogen and stored at −80 °C until used.

B.2. Crystallisation

Cubic bovine insulin crystals suitable for the generation of seeds were grown in Greiner CrystalQuick™, 96-well sitting drop plates with a reservoir volume of 90 µL and a final drop of 400 nL; 1:1 protein to precipitant solution. Insulin solution was mixed with a reservoir solution; 0.1 M BisTris Propane pH 7.5, 0.1 M KI and 22 % (w/v) PEG 3,350. The same protocol was used to create thermolysin seed crystals but with a reservoir solution of 22 % (w/v) PEG 3,350, and 0.2 M ammonium chloride and 20 % (w/v) PEG 3,350. After approximately 24 hr the crystals could be harvested for seeding purposes. Crystals from 5 drops were pooled in 250 µL of crystallisation buffer and ten to fifteen 1 mm glass milled beads (Fisher Scientific) were added to this solution. The solution was then alternately vortexed and cooled on ice every 30 s from three cycles. This stock was then frozen and stored at −20 °C until required.

[2] https://www.oppf.rc-harwell.ac.uk/OPPF/protocols/popin/gb/pOPINF.gb

Proteinase K crystals were grown in a 24-well sitting drop plate (Mitegen). Proteinase K solution was mixed in a 1:1 ratio with 0.2 M NH_4Cl, 20 % (w/v) PEG 3,350 to a final volume of 10 µL. Crystals appeared after 6 hr and grew for a further 24 at 20 °C.

B.3. Scanning electron microscopy

Samples were cryo-cooled on glow-discharged Cu 200 mesh Quantifoil™ R 2/2 electron microscopy grids (Quantifoil). First, a 2 µL aliquot of well solution was applied to the Cu side of the grid, then 2 µL of the slurry of crystals was applied onto the carbon film. The grid was then blotted for 4.0 s from the Cu side of the grid using a Leica EM GP plunge freezer. During this process, the chamber environment of the Leica EM GP was set to 20 °C and 90 % humidity. After blotting, grids were immediately plunge frozen in liquid ethane and then transferred to liquid nitrogen for storage until further use.

SEM images were taken using a JEOL JSM-IT100 SEM equipped with a Quorum PP3000T cryostage and cryotransfer system. The PP3000T cryostage, preparation stage and anticontaminator were cooled to −180 °C, −180 °C and −190 °C, respectively. The crystal seeds were imaged using an accelerating voltage of 5 kV and a beam current of 47 pA.
